# An ordered assembly of MYH glycosylase, SIRT6 protein deacetylase, and Rad9-Rad1-Hus1 checkpoint clamp at oxidatively damaged telomeres

**DOI:** 10.18632/aging.103934

**Published:** 2020-09-29

**Authors:** Jun Tan, Xiangyu Wang, Bor-Jang Hwang, Rex Gonzales, Olivia Konen, Li Lan, A-Lien Lu

**Affiliations:** 1 Massachusetts General Hospital Cancer Center, Department of Radiation Oncology, Harvard Medical School, Charlestown, MA 02129, USA; 2 Department of Biochemistry and Molecular Biology, University of Maryland School of Medicine, Baltimore, MD 21201, USA; 3 Nathan Schnaper Intern Program in Translational Cancer Research, University of Maryland School of Medicine, 108 North Greene Street, Baltimore, MD 21201, USA; 4 Marlene and Stewart Greenebaum Comprehensive Cancer Center, University of Maryland School of Medicine, Baltimore, MD 21201, USA

**Keywords:** SIRT6, MutY homolog (MYH or MUTYH), checkpoint clamp Rad9/Rad1/Hus1 (9-1-1), DNA damage response, telomeres

## Abstract

In the base excision repair pathway, MYH/MUTYH DNA glycosylase prevents mutations by removing adenine mispaired with 8-oxoG, a frequent oxidative lesion. MYH glycosylase activity is enhanced by Rad9-Rad1-Hus1 (9-1-1) checkpoint clamp and SIRT6 histone/protein deacetylase. Here, we show that MYH, SIRT6, and 9-1-1 are recruited to confined oxidatively damaged regions on telomeres in mammalian cells. Using different knockout cells, we show that SIRT6 responds to damaged telomeres very early, and then recruits MYH and Hus1 following oxidative stress. However, the recruitment of Hus1 to damaged telomeres is partially dependent on SIRT6. The catalytic activities of SIRT6 are not important for SIRT6 response but are essential for MYH recruitment to damaged telomeres. Compared to wild-type MYH, the recruitment of hMYH^V315A^ mutant (defective in both SIRT6 and Hus1 interactions), but not hMYH^Q324H^ mutant (defective in Hus1 interaction only), to damaged telomeres is severely reduced. The formation of MYH/SIRT6/9-1-1 complex is of biological significance as interrupting their interactions can increase cell’s sensitivity to H_2_O_2_ and/or elevate cellular 8-oxoG levels after H_2_O_2_ treatment. Our results establish that SIRT6 acts as an early sensor of BER enzymes and both SIRT6 and 9-1-1 serve critical roles in DNA repair to maintain telomere integrity.

## INTRODUCTION

Genomic integrity is threatened by reactive oxygen species (ROS) that are produced as normal metabolic byproducts and by exposure to external reagents or radiation [1]. Oxidative damage to DNA has been implicated in aging, neurodegenerative diseases, and cancer [2]. Particularly, the C-G-rich telomeres are highly susceptible to oxidative damage [3, 4]. 8-oxo-7,8-dihydroguanine (8-oxoG, G^o^) is a frequent and highly mutagenic oxidative lesion [5]. Approximately 10^3^ and 10^5^ G^o^ lesions/cell/day are found in normal and cancer tissues, respectively [5]. If not repaired, G^o^ mispairs with adenine during DNA replication resulting in a G:C to T:A mutation [6–8]. Oxidative DNA lesions are repaired primarily by the base excision repair (BER) pathway [9]. In mammalian cells, the misincorporated adenines in A/G^o^ mismatches are removed by the MutY homolog (MYH or MUTYH)-directed BER pathway [6, 10, 11]. After the action of MYH glycosylase, apurinic/apyrimidinic endonuclease 1 (APE1) recognizes and cleaves the abasic site and other enzymes complete the repair process in the long-patch BER pathway [12]. Mutations in the human *MYH* (*hMYH*) gene can lead to colorectal cancer (as in MYH-associated polyposis or MAP) [13]. APE1 is essential for cell viability [14] and telomere maintenance [15].

DNA damage response (DDR) coordinates DNA repair with other cellular processes in eukaryotic cells [16]. Cell cycle checkpoints provide surveillance mechanisms to activate DDR [16, 17] which in turn elicits both DNA repair processes and cell cycle arrest, thus allowing time for DNA repair. When DNA damage is extreme, apoptosis is triggered. In DDR, the checkpoint clamp Rad9-Rad1-Hus1 (9-1-1) is required to activate ATR protein kinase which then phosphorylates downstream proteins [18, 19]. In addition to its role in DDR [20, 21], 9-1-1 is directly involved in many DNA transactions including BER (reviewed in [22, 23]). It has been suggested that 9-1-1 provides a platform to coordinate BER processes because it interacts with and stimulates nearly every enzyme in BER [23]. 9-1-1 is essential for embryonic development, genomic stability, and telomere integrity [22, 24–28].

DNA repair also requires chromatin remodeling. The aging regulator SIRT6 is a NAD^+^-dependent histone/protein deacetylase (reviewed in [29]) and also has mono-ADP-ribosyltransferase and defatty-acylase (protein lysine fatty acyl removal) activities [29, 30]. SIRT6 has roles in stress response, DNA repair, telomere integrity, retrotransposition, and metabolic homeostasis [29, 31–36]. Importantly, SIRT6 overexpression extends lifespan [37] and its depletion leads to premature cellular senescence [31, 32, 38]. SIRT6 can recruit SNF2H (one of the ISWI chromatin remodeling complexes) to DNA break sites [34] and to oxidatively damaged telomeres [39], leading to locally decondensed chromatin. SIRT6 plays a direct role in BER and DNA damage response through physical interactions and functional stimulation [40–43]. Particularly, SIRT6 directly interacts with MYH, APE1, and 9-1-1; and stimulates MYH and APE1 activities, thus connecting chromatin remodeling and MYH-directed BER [40].

Telomeres protect the ends of each chromosome from deterioration and fusion [44]. Telomere dysfunction can lead to aging-related degenerative pathologies and cancer [45]. Mammalian telomeric DNA contains long tandem (TTAGGG) repeats which are highly susceptible to oxidative damage [3, 4, 46–48]. Oxidative damage to telomeric DNA accelerates telomere shortening and affects telomere integrity [3, 4]. The presence of G^o^ in telomeric DNA disrupts telomerase activity [49] and inhibits the binding of protein factors [50]. It has been shown that targeted and persistent G^o^ at telomeres promotes telomere shortening, aberration, and crisis [51, 52]. Therefore, telomeres are reliant upon efficient DNA repair to maintain their integrity. Several reports suggest that telomere stability requires the repair of oxidized bases at telomeres [47, 48, 53]. Biochemical studies show that telomere binding proteins interact with many BER proteins and stimulate their activities [53, 54]. 9-1-1 is associated with telomeres and is essential for telomere stability [22, 24]. SIRT6 modulates histone acetylation levels at telomeric chromatin and regulates telomere function [31, 32]. APE1 plays an essential role in telomere maintenance [15]. We have also shown that hMYH and hSIRT6 are associated with telomeres, and mouse Myh (mMyh) foci are induced on telomeres by oxidative stress [40]. These results highlight the importance of the roles of 9-1-1, SIRT6, and BER in telomere maintenance.

We have shown that MYH, SIRT6, and 9-1-1 form a complex to maintain genomic and telomeric integrity in mammalian cells [40]. Interestingly, SIRT6 and Hus1 bind to the interdomain connector (IDC, residues 295-350) [40, 55] located between the N- and C-terminal domains of hMYH, but they do not compete for MYH association [56]. We also show that MYH and SIRT6 are efficiently recruited to KillerRed (KR)-induced confined oxidative DNA damage sites within transcriptionally active chromatin, but not to the DNA damage sites within inactive chromatin [40]. Upon activation by 550–580 nm light, KR releases localized superoxide which may produce clustered oxidative base damage, leading to single-stranded and double-stranded DNA break production and telomere loss [52]. To investigate how MYH, SIRT6, and 9-1-1 are recruited to damaged telomeric DNA in mammalian cells, we used a novel fluorescence technique [52] to directly examine their ordered assembly at telomeres in different knockout (KO) cells. We show that SIRT6 responds to damaged telomeres very early and then recruits MYH and Hus1. However, the recruitment of Hus1 to the damaged telomeres is partially dependent on SIRT6. Interestingly, the catalytic activities of SIRT6 are not important for SIRT6 response but are essential for MYH recruitment to damaged telomeres. Because the formation of the MYH/SIRT6/ 9-1-1 complex occurs in a cooperative manner, the response of one component to DNA damage is affected when the other partner is absent. For example, even when MYH interacts with SIRT6, its foci formation at damaged telomeres is abolished in *hus1* KO cells. We also show that interrupting MYH interactions with its partners by expressing wild-type IDC (IDC-WT) peptide can increase cell’s sensitivity to H_2_O_2_ and elevate cellular 8-oxoG levels after H_2_O_2_ treatment. Our results indicate interactions of MYH with Hus1, SIRT6 and APE1 are important in controlling cell viability and that MYH-Hus1 interaction is critical in reducing 8-oxoG levels.

## RESULTS

### MYH, SIRT6, Hus1, and Rad9 are associated with oxidatively damaged telomeric chromatin

Although MYH, SIRT6, and 9-1-1 are all telomeric associated proteins [22, 24, 31, 32, 40], their responses to DNA damage are not clear. To investigate how MYH, 9-1-1, and SIRT6 respond to oxidatively damaged telomeres, we employed our newly developed inducible-ROS systems to confine DNA damage to telomeric chromatin [52]. In this method, local oxidative DNA damage within telomeric DNA is induced by activating KillerRed (KR) protein fused to telomeric repeat binding protein TRF1. Upon visible light illumination (550–580 nm), the photosensitizer KillerRed releases superoxide which may produce clustered oxidative base lesions leading to the production of single-stranded and double-stranded DNA breaks [52, 57]. Thus, after light induction, KR-TRF1 fusion protein bound to the telomeric TTAGGG repeat sequence can induce localized DNA damage. This novel approach allows us to study, on fine scale, exactly how the BER complex is assembled at lesion sites in live mammalian cells.

**Figure 1:**
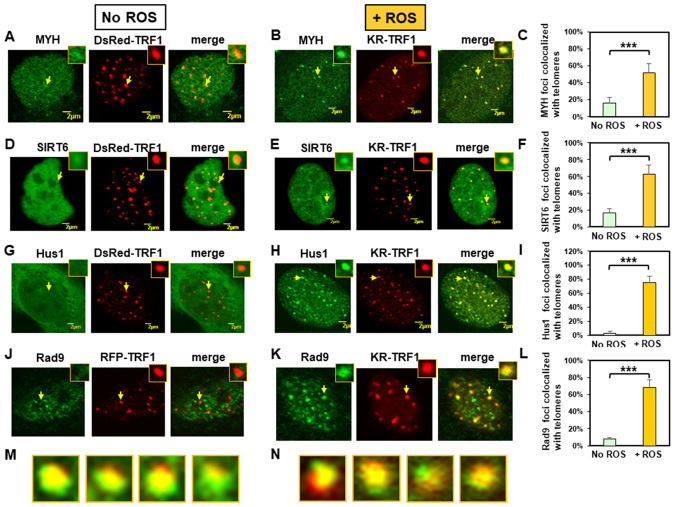
**Expressed MYH, SIRT6, Hus1, and Rad9 are recruited to oxidatively damaged telomeric sites in mouse embryonic fibroblast (MEF) cells.** (**A**), (**D**), (**G**), and (**J**), Distribution of GFP-hMYH, GFP-hSIRT6, GFP-hHus1, and FLAG-Rad9, respectively, in undamaged MEF cells containing DsRed-TRF1 or RFP-TRF1. Some GFP-hMYH and GFP-hSIRT6 granules are localized to DsRed-TRF1 at telomeres as shown in enlarged merged images. (**B**), (**E**), (**H**), and (**K**), GFP-hMYH, GFP-hSIRT6, GFP-hHus1, and FLAG-Rad9, respectively, form foci at KR-TRF1 damaged telomeric sites after light activation. Images were captured 30 min after light activation with an Olympus FV1000 confocal microscopy system. (**C**), (**F**), (**I**), and (**L**), Quantitative analyses of 20 cells in each undamaged and KR-induced damaged group. About 15% of granulated GFP-MYH and GFP-SIRT6 spots were localized to DsRed-TRF1 at telomeres (**C**, **F**). Over 50% of GFP-MYH, GFP-SIRT6, or GFP-Hus1 foci are colocalized with KR-TRF1 after KR activation. Error bars indicate SD; n ≥ 20. The *P*-value is calculated by Student’s t-test using Stat Plus software; *P <* 0.01 is shown as ***. (**M**), Enlarged merged images from (**H**) showing segregation of green GFP-hHus1 and red KR-TRF1 foci. (**N**), Enlarged merged images from (**K**) showing segregation of green FLAG-hRad9 and red KR-TRF1 foci.

We analyzed ectopically expressed GFP-tagged MYH, SIRT6, and Hus1 in MEF cells expressing either KR-TRF1 or a non-phototoxic red fluorescent protein (DsRed)-tagged TRF1 (DsRed-TRF1). First, we examined whether GFP-tagged proteins were biologically active. GFP-tagged hMYH and hSIRT6 proteins were active because they could reduce 8-oxoG levels in the respective knockout cells following peroxide treatment (see Supplementary Figures 1A and 1B). Expression of GFP-hHus1 protein could reduce the sensitivity of *hus1* KO cells to hydroxyurea (a DNA replication blocker) (Supplementary Figure 1C). In addition, *MYH*, *sirt6*, and *hus1* KO cells expressing GFP-MYH, GFP-SIRT6, GFP-Hus1, respectively, contained fewer apoptotic cells than vector-transfected KO cells (Supplementary Figure 1D–1F). This is the first demonstration that SIRT6 is important in preventing 8-oxoG accumulation and apoptosis following oxidative stress. In cells expressing DsRed-TRF1, both GFP-MYH and GFP-SIRT6 appeared granulated in faint spots throughout the nucleoplasm of MEF cells (Figure 1A and 1D). Interestingly, about 15% of granulated GFP-MYH and GFP-SIRT6 spots were localized to DsRed-TRF1 at telomeres, as seen in the merged images of 20 cells from each group (Figure 1C and 1F). In undamaged cells, GFP-tagged Hus1 was localized mainly in cytoplasm and only about 3% of Hus1 foci colocalized with telomeres (Figure 1G and 1I). In cells transfected with KR-TRF1 without light induction (Supplementary Figure 2), the response and distribution of MYH, SIRT6, and Hus1 were essentially the same as cells with DsRed-TRF1 (Figure 1). Before light activation of KR, 11-14% of GFP-MYH, GFP-SIRT6, or endogenous mMyh granules were localized to telomeres (Supplementary Figure 2A, 2B, 2E, and 2G), while only about 3-7% of GFP-Hus1, FLAG-Rad9, or endogenous mHus1 granules were colocalized with telomeres (Supplementary Figure 2C, 2D, 2F, and 2G).

We then performed the response kinetics of GFP-proteins to DNA damage at telomeres. As shown in Supplementary Figure 3, 30 min was the maximum time point for the damage response to occur. After ROS induction by activating the KR protein, GFP-tagged MYH, SIRT6, and Hus1 all formed discrete nuclear foci which colocalized with telomeres (Figure 1B, 1E, and 1H, yellow foci in the merged images). Because MYH removes adenine misinserted opposite 8-oxoguanine following DNA replication, we reason that some unsynchronized MEF cell populations are at G1- or S-phase. Therefore, some cells analyzed at 30 min after KR-induction are at S-phase and are replicating their DNA. In addition, MYH also binds to cytosine paired with 8-oxoG with high affinity [58, 59]. In this case, MYH can form foci at telomeres at any cell cycle stage. By analyzing 20 cells from each group, over 50% of GFP-MYH, GFP-SIRT6, or GFP-Hus1 foci exhibited colocalization with KR-TRF1 after KR activation (Figures 1C, 1F, and 1I). The colocalization of GFP-MYH, GFP-SIRT6 or GFP-Hus1 foci at sites of telomeres after light activation of KR was significantly increased compared to before light activation (Supplementary Figure 2). Using immunofluorescence staining, we also showed that FLAG-tagged Rad9 (one subunit of 9-1-1) acted similarly to Hus1 in the damage response (Figure 1J, 1K and 1L). However, in undamaged cells, about 8% of Rad9 foci colocalized with telomeres (Figure 1J and 1L). The FLAG-Rad9 protein has been shown to be active by rescuing the homologous recombination defect caused by *Rad9* knockdown [60]. Interestingly, about 40% of Hus1 and Rad9 foci at telomeres expanded unidirectionally from KR-TRF1 sites as seen in the enlarged images (Figure 1M and 1N). Since nuclear Hus1 is likely to form a complex with Rad9, and Rad1 [61], and is the partner of MYH and SIRT6 [40, 55], Hus1 staining is used to represent the entire 9-1-1 complex in the nucleus.

We have shown that the endogenous SIRT6 and tagged SIRT6 proteins are recruited similarly to telomeres [39]. To confirm that endogenous mMyh and mHus1 proteins behave in a similar manner as tagged proteins in live cells, we proceeded with immunofluorescence staining to determine their response to DNA damage at telomeres. As shown in Supplementary Figure 4, the endogenous mMyh and mHus1 behaved in a similar manner as GFP-tagged proteins in regard to their colocalization with telomeres. About 20% of granulated mMyh spots were localized to RFP-TRF1 at telomeres, as seen in the merged images of 20 cells from each group (Supplementary Figure 4C). In undamaged cells, only 7% of mHus1 foci colocalized with telomeres (Supplementary Figure 4D and 4F). After ROS induction by activating the KR protein, mMyh and mHus1 formed discrete nuclear foci (Supplementary Figure 4B and 4E, yellow foci in the merged images). By analyzing 20 cells from each group, over 80% of mMyh or mHus1 foci showed colocalization with KR-TRF1 after KR activation (Supplementary Figure 4C and 4F). In summary, our data suggest that MYH, SIRT6, and 9-1-1 are recruited to telomeric chromatin to repair oxidative DNA damages.

### MYH foci induced at oxidatively damaged telomeres are dependent on Hus1 while Hus1 foci formation at damaged telomeres is independent of MYH

**Figure 2:**
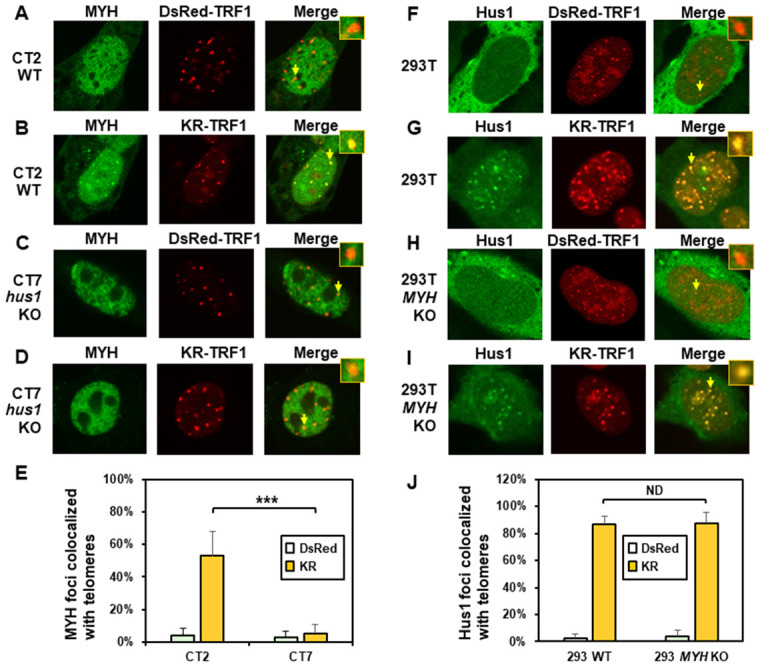
**The formation of MYH foci induced at oxidatively damaged telomeres is dependent on Hus1.** (**A** and **C**) GFP-MYH does not form foci at sites with DsRed-TRF1 in undamaged CT2 (*hus1^+^*^/+^) and CT7 (*hus1*^-/-^ *p21*^-/-^) MEF cells, respectively. These cells contain granulated GFP-MYH spots and some of spots were localized with telomeres. (**B** and **D**) Damage response of GFP-MYH to the sites of KR-TRF1 after light activation in CT2 and CT7 MEF cells, respectively. GFP-MYH foci are not found at the sites of KR-TRF1 in CT7 cells. (**E**), Analyses of about 20 cells in each (**A**–**D**) group indicated that approximately 50% of GFP-MYH foci colocalized with telomeres in CT2 cells, in contrast, less than 5% of GFP-MYH foci colocalized with telomeres in CT7 cells. (**F**–**J**), GFP-Hus1 foci formation at telomeres in control HEK-293T and *MYH* KO HEK-293T human cells. Experiments were performed similarly to (**A**–**E**) except using GFP-hHus1 and different cells. After ROS induction by activating the KR protein, over 80% of GFP-Hus1 foci are colocalized with KR-TRF1 in both HEK-293T and *MYH* KO HEK-293T cells (**J**). ND indicates no difference.

To examine the mutual dependence of MYH and 9-1-1 association at DNA damage sites, we used the KR-TRF1 system in conjunction with cell knockout approaches. We transfected both CT2 (*hus1^+^*^/+^) and CT7 (*hus1*^-/-^ *p21*^-/-^) MEF cells with both KR-TRF1 (or DsRed-TRF1 as controls) and GFP-hMYH. As observed in Figure 2A, undamaged CT2 and CT7 cells contained granulated GFP-MYH spots and some of spots were localized with telomeres (Figure 2A and 2C). After ROS induction by activating the KR protein, GFP-hMYH formed discrete nuclear foci, of which 50% colocalized with KR-TRF1 marked telomeres in CT2 cells (Figure 2B and 2E). In contrast, GFP-MYH did not form foci in CT7 cells. Thus, the formation of MYH foci at damaged telomeres is dependent on Hus1.

Next, we examined GFP-Hus1 foci formation at telomeres in KR-TRF1-transfected MYH KO cells. For this purpose, the *hMYH* gene in human HEK-293T cells was knocked out by CRISPR-Cas9 method [62]. After transfection, with MYH crRNA 2 plasmid colonies were selected with 1.25 μg/ml puromycin and single colonies were screened, expanded, and confirmed by DNA sequencing and Western blot analysis. *MYH* KO clone 7a did not contain hMYH proteins in Western blotting (Supplementary Figure 5A). PCR and DNA sequencing analyses indicated that one allele of the *hMYH* gene contained an adenine insertion and the other allele contained a 229-bp deletion in *MYH* KO clone 7a. The majority of Hus1 were in cytoplasm (Figure 2F and 2H) and very few granulated GFP-Hus1 spots were colocalized with telomeres (Figure 2J) in undamaged control HEK-293T and *MYH* KO cells. After ROS induction by activating the KR protein, GFP-Hus1 formed discrete nuclear foci of which about 90% colocalized with KR-TRF1 marked telomeres in both HEK-293T and *MYH* KO HEK-293T cells (Figure 2G, 2I, and 2J). Thus, Hus1 foci formation at damaged telomeres is independent of MYH.

### MYH foci induced at oxidatively damaged telomeres are dependent on SIRT6 while SIRT6 foci formation at damaged telomeres is independent of MYH

**Figure 3:**
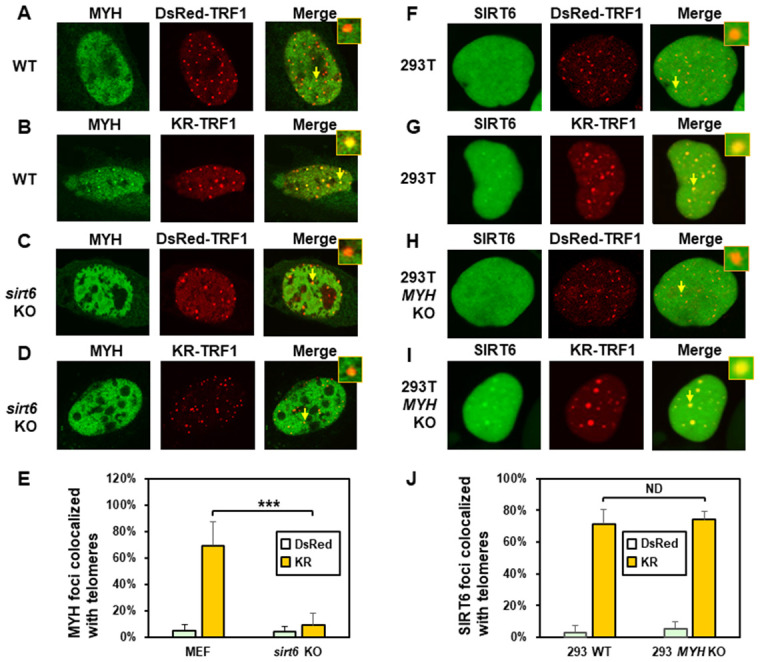
**The formation of MYH foci induced at oxidatively damaged telomeres is dependent on SIRT6.** (**A** and **C**), GFP-MYH does not form foci at sites with DsRed-TRF1 in undamaged control MEF and *sirt6* KO MEF cells, respectively. (**B** and **D**), Damage response of GFP-MYH to the sites of KR-TRF1 after light activation in control and *sirt6* KO MEF cells, respectively. GFP-MYH foci were not found at the sites of KR-TRF1 in *sirt6* KO cells. (**E**), Analyses of about 20 cells in each (**A**–**D**) group indicated that approximately 70% of GFP-MYH foci colocalized with telomeres in control cells, in contrast, less than 10% of GFP-MYH foci colocalized with telomeres in *sirt6* KO cells. (**F**–**J**), GFP-hSIRT6 foci formation at telomeres in control HEK-293T and *MYH* KO HEK-293T human cells. Experiments were performed similarly to (**A**–**E**) except using GFP-hSIRT6 and different cells. After ROS induction by activating the KR protein, about 70% of GFP-SIRT6 foci are colocalized with KR-TRF1 in both HEK-293T and *MYH* KO cells (**J**).

Next, we compared MYH response to KR-TRF1 induced telomeric damage in wild-type (WT) and *sirt6* KO (*sirt6^-/-^)* MEF cells. Undamaged WT and *sirt6^-/-^* MEF cells contained GFP-MYH granulated spots, some of which were localized to telomeres (Figure 3A and 3C). After ROS induction by activating the KR protein, about 70% of GFP-hMYH foci colocalized with telomeres in WT MEF cells (Figure 3B and 3E). Importantly, GFP-MYH foci formation was substantially reduced in damaged *sirt6^-/-^* cells (Figure 3D and 3E).

We then examined SIRT6 foci formation at KR-TRF1-induced damaged telomeres in HEK-293T and *MYH* KO HEK-293T cells. There were some granulated GFP-SIRT6 spots in both undamaged cells (Figure 3F and 3H). It appeared that more granulated GFP-SIRT6 spots were in undamaged *MYH* KO cells than control HEK-293T cells, although this was not statistically significant (Figure 3J). After ROS induction by activating the KR protein, GFP-SIRT6 formed discrete nuclear foci of which about 70% colocalized with telomeres in both HEK-293T and *MYH* KO cells (Figure 3G, 3I, and 3J). In summary, MYH foci formation at damaged telomeres is dependent on SIRT6, but SIRT6 recruitment to damaged telomeres is independent of MYH.

### The recruitment of Hus1 to damaged telomeres is partially dependent on SIRT6 while SIRT6 recruitment to damaged telomeres is independent of Hus1

**Figure 4:**
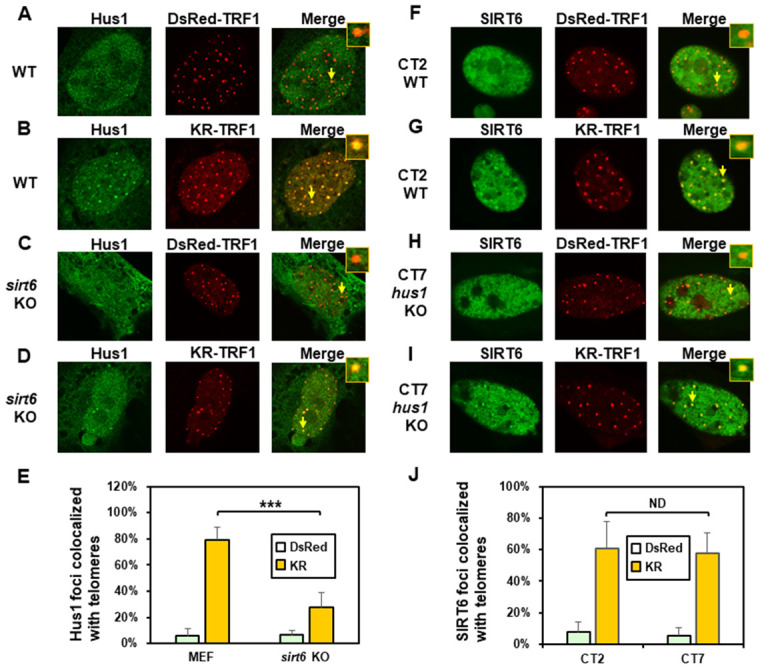
**The formation of Hus1 foci induced at oxidatively damaged telomeres is partially dependent on SIRT6.** (**A** and **C**), GFP-Hus1 does not form foci at sites with DsRed-TRF1 in undamaged control MEF and *sirt6* KO MEF cells, respectively. (**B** and **D**), Damage response of GFP-Hus1 to the sites of KR-TRF1 after light activation in control and *sirt6* KO MEF cells, respectively. GFP-hHus1 foci are significantly reduced at the sites of KR-TRF1 in *sirt6* KO cells. (**E**), Analyses of about 20 cells in each (**A**–**D**) group indicated that approximately 80% of GFP-Hus1 foci colocalized with telomeres in control cells, in contrast, only 30% of GFP-Hus1 foci colocalized with telomeres in *sirt6* KO cells. (**F**–**J**), GFP-hSIRT6 foci formation at telomeres in control CT2 and *Hus1* KO CT7 MEF cells. Experiments were performed similarly to (**A**–**E**) except using GFP-hSIRT6 and different cells. After ROS induction by activating the KR protein, about 60% of GFP-SIRT6 foci are colocalized with KR-TRF1 in both CT2 and CT7 cells (**J**).

We also determined the order of Hus1 and SIRT6 association at damaged telomeres. As expected, about 80% of GFP-Hus1 foci colocalized with telomeres in WT MEF cells after ROS induction (Figure 4B and 4E). In undamaged *sirt6^-/-^* MEF cells, Hus1 was present in both the cytoplasm and nucleus (Figure 4C). GFP-Hus1 did form foci at damaged telomeres in damaged *sirt6^-/-^* MEF cells, however, only about 30% of Hus1 foci colocalized with KR-TRF1 marked telomeres (Figure 4D and 4E, yellow foci in the merged image) and some telomeres did not contain Hus1 foci (Figure 4D, red foci in the merged image). Therefore, Hus1 foci formation at damaged telomeres is partially dependent on SIRT6. We then examined SIRT6 foci formation at KR-TRF1-induced damaged telomeres in CT2 (*hus1*^+/+^) and CT7 (*hus1*^-/-^) cells. The responses of SIRT6 to damaged telomeres were similar in both cell lines (Figure 4F–4I). As shown in Figure 4J, about 60% of GFP-SIRT6 foci colocalized with telomeres in both WT and *hus1* KO MEF cells after ROS induction. Thus, SIRT6 foci formation at damaged telomeres is independent of Hus1.

### SIRT6 enzymatic activities are not important for SIRT6 response but are necessary for the recruitment of MYH to damaged telomeres

**Figure 5:**
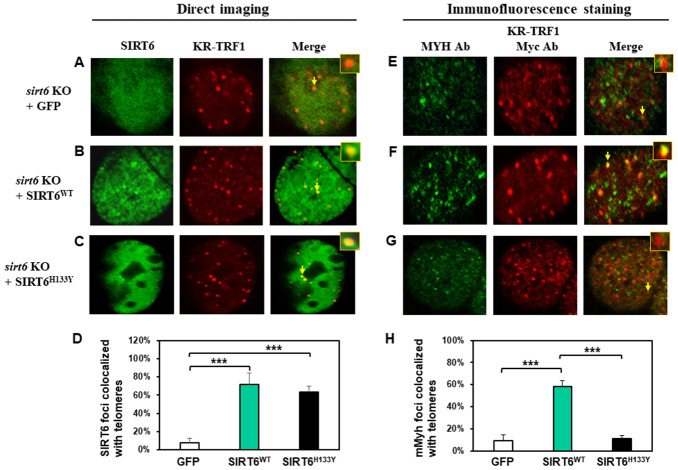
**The recruitment of MYH at oxidatively damaged telomeres is dependent on the catalytic activities of SIRT6.** MEF *sirt6* KO cells were transfected with pEGFP-C1 vector or vector containing human wild-type (WT) or H133Y mutant *hSIRT6* gene along with KR-TRF1 plasmid. (**A**–**D**), Both GFP-tagged hSIRT6^WT^ and hSIRT6^H133Y^ respond similarly to damaged telomeres. GFP-SIRT6 and Myc-tagged KR-TRF1 were detected by direct imaging. Images were captured 30 min after light activation with an Olympus FV1000 confocal microscopy system. (**E**–**H**), Response of endogenous mMyh to the damage sites in *sirt6* KO cells expressing hSIRT6^WT^ or hSIRT6^H133Y^ mutant. Cell fluorescence was stripped by HCl treatment. GFP-SIRT6, MYH, and Myc-KR-TRF1 were them detected by immunofluorescence staining with GFP, MYH, and Myc antibodies (Ab), respectively, and reacted with blue, green, and red emission secondary antibodies, respectively. Cells containing blue-colored GFP-SIRT6 were selected for analyses to detect mMyh green foci and red-colored telomeres. (**D**) and (**H**), Quantitative analyses of 20 cells as in (**A**–**C**) and (**E**–**F**) groups, respectively. White, green, and black bars represent protein colocalization with telomeres in MEF *sirt6* KO cells containing GFP alone, GFP-hSIRT6^WT^, or GFP-hSIRT6^H133Y^, respectively.

It has been shown that both protein deacetylase and ADP-ribosylase activities of SIRT6 facilitate DNA repair [63]. Since SIRT6 is required for MYH recruitment to damaged telomeres, we examined whether its catalytic activities were necessary for this function. We tested whether SIRT6^H133Y^ mutation could affect the recruitment of MYH to damaged telomeres. SIRT6^H133Y^ mutant has been shown to lack both NAD^+^-dependent protein deacetylation and ADP-ribosylation [38, 41] while also having very weak defatty-acylase activity [64]. MEF *sirt6* KO cells were transfected with vector or vector containing wild-type (WT) or H133Y mutant *hSIRT6* gene along with KR-TRF1 plasmid. As shown in Figure 5B–5D, direct imaging showed that similar percentages of hSIRT6^WT^ and hSIRT6^H133Y^ were colocalized with damaged telomeres. This indicates that the catalytic activities of SIRT6 are not necessary for SIRT6 response to damaged sites. Because the transfection frequencies with three plasmids into MEF cells are very low, we selected to examine endogenous mMyh in MEF cells transfected with two plasmids. We first tried to detect endogenous mMyh by immunofluorescence staining with blue emission secondary antibody in MEF cells containing GFP-SIRT6 and KR-TRF1. However, we were unable to clearly define blue mMyh foci because their signal was obscure and also interfered by the green and red fluorescence. We modified the method by stripping all fluorescence by HCl treatment after KR activation. mMyh, GFP-SIRT6, and Myc-KR-TRF1 were then detected by MYH, GFP, and Myc antibodies which were reacted with green, blue, and red emission secondary antibodies, respectively. Cells containing blue-colored GFP-SIRT6 were selected for analyses of the colocalization of mMyh green foci and red-colored telomeres (Figure 5E–5G). Approximately 9% of mMyh foci were observed in damaged *sirt6^-/-^* cells transfected with vector alone (Figure 5E and 5H). This result is similar to that with GFP-MYH in *sirt6^-/-^* cells (Figure 3E). After ROS induction by activating the KR protein, approximately 60% of mMyh foci were colocalized with KR-TRF1 in MEF *sirt6* KO cells expressing hSIRT6^WT^ protein (Figure 5F and 5H). Interestingly, the telomere colocalizations of mMyh was absent in *sirt6* KO cells expressing hSIRT6^H133Y^ mutant (Figure 5G and 5H). This result indicates that the activities of SIRT6 are essential for MYH recruitment to damaged telomeres.

### Mutations in the interdomain connector of MYH influence MYH association with damaged telomeres

**Figure 6:**
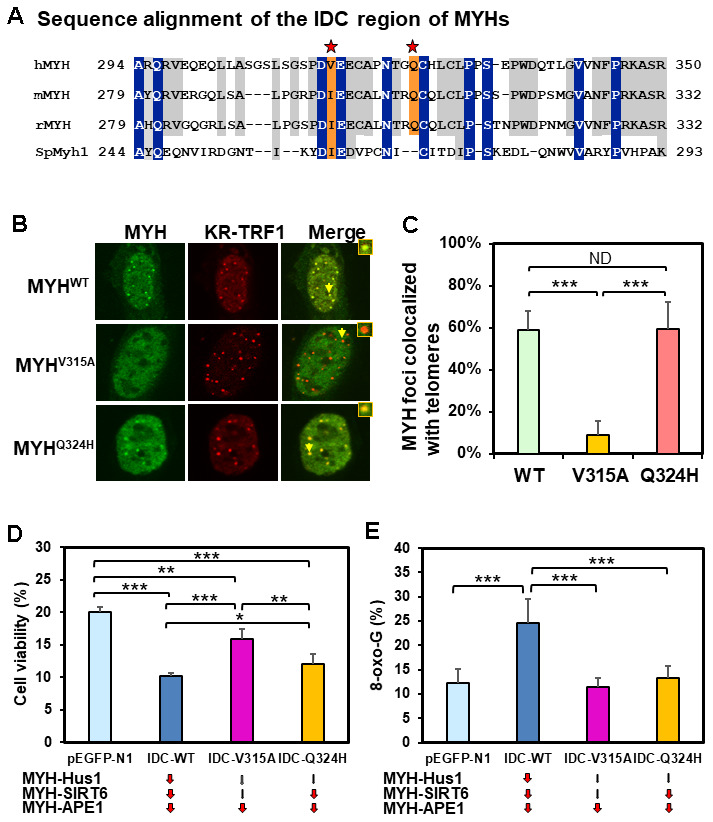
**Human V315A mutant MYH protein expressed in mouse cells cannot associate with damaged telomeres and interrupting the MYH interactions with its partners can increase cell’s sensitivity to H_2_O_2_ and/or elevate cellular 8-oxoG levels.** (**A**) Sequence alignment of the IDC regions of eukaryotic MYH proteins. V315 and Q324 of hMYH are important for interaction with Hus1 [55, 66] (red stars). However, residue Q324 of hMYH is dispensable for interacting with SIRT6 [40]. (**B**) and (**C**) GFP-MYH^WT^, GFP-MYH^V315A^, and GFP-MYH^Q324H^ along with KR-TRF1 were expressed in MEF cells to determine their association with damaged telomeres. The association of GFP-MYH^V315A^, but not GFP-MYH^Q324H^, with damaged telomeres is substantially attenuated. (**D**) HEK-293T cells transformed with pEGFP-N1 vector or vector with IDC sequences were treated with 700 mM H_2_O_2_ for 1 h and recovered in fresh medium for two days. Cell viability was measured as described in Materials and Methods. (**E**) HEK-293T cells transformed with pEGFP-N1 vector or vector with IDC sequences were treated with 700 mM H_2_O_2_ for 1 h and recovered in fresh medium for 1 h. 8-oxoG levels were measured. Each average value was obtained by subtracting the value of cells containing pEGFP-N1 vector without H_2_O_2_ treatment from H_2_O_2_-treated cells with listed plasmids. *, **, and *** represent *P* <0.1, *P* <0.05, and *P* < 0.01, respectively.

The IDC of MYH has a unique architecture [56] that serves as a hub for interactions with Hus1, SIRT6, and APE1 [40, 55, 65]. V315 and Q324 of hMYH are important for interaction with Hus1 [55, 66] (red stars in Figure 6A). However, residue Q324 of hMYH is dispensable for interaction with SIRT6 [40]. To determine how these residues control MYH association with damaged telomeres, we expressed GFP-MYH^WT^, GFP-MYH^V315A^, or GFP-MYH^Q324H^ along with KR-TRF1 in CT2 MEF cells. The results (Figure 6B and 6C) demonstrated that the association of GFP-MYH^V315A^ (but not GFP-MYH^Q324H^) with damaged telomeres was substantially attenuated. Taken together with the results, and the deficiency of MYH mutants in protein-protein interactions, SIRT6 is an essential partner for MYH association with damaged telomeres.

### Human cells expressing GFP-IDC peptides have increased sensitivity to H_2_O_2_ and/or elevated 8-oxoG levels

The IDC of MYH interacts with Hus1, SIRT6, and APE1 [40, 55, 65]. We hypothesized that IDC overproduction might inhibit MYH interactions with its partners and diminish BER. We expressed GFP-tagged hMYH-IDC peptides containing WT, V315A mutant, and Q324H mutant sequences in HEK-293T cells. IDC-WT expression is expected to impede MYH interaction with Hus1, SIRT6, and APE1; IDC-V315A expression is expected to interfere with MYH interaction with APE1, but not as much with Hus1 and SIRT6; IDC-Q324H expression is expected to block MYH interaction with APE1 and SIRT6, but not with Hus1. After H_2_O_2_ treatment, cell viability and 8-oxoG levels were measured. As indicated in Figure 6D and 6E, cells expressing IDC-WT peptide had increased sensitivity to H_2_O_2_ and elevated 8-oxoG levels as compared to cells transfected with vector alone. Cells expressing IDC-V315A peptide had slightly increased sensitivity to H_2_O_2_, but the 8-oxoG levels were the same as cells transfected with vector. Cells expressing IDC-Q324H peptide had significantly increased sensitivity to H_2_O_2_, while the 8-oxoG levels were slightly increased when compared to cells transfected with vector alone, but this increase was statistically insignificant. Thus, interrupting the MYH interactions with its partners can increase cell’s sensitivity to H_2_O_2_ and/or elevate cellular 8-oxoG levels after H_2_O_2_ treatment.

## DISCUSSION

Mammalian chromosomal ends resemble double-stranded DNA breaks, but they do not activate a damage response to DNA strand breaks in healthy cells [67]. The Shelterin components prevent the recognition of telomeres as sites of double-stranded DNA damage [68]. However, oxidative damages frequently occur on the G-rich telomeric DNA [3, 4, 46–48] and this affects telomere integrity [3, 4, 51]. Therefore, telomeres require efficient BER and proper DDR to maintain their integrity [47, 48, 53]. The KR-TRF1 inducible system may produce multiple types of DNA damage, including base lesions, single-strand breaks, and double-strand breaks [52]. It has been shown that several BER factors (NTH1, Polβ, and FEN1) are recruited to oxidatively damaged telomeres using the KR-TRF1 system [52], suggesting that BER is responsible for oxidative damage repair. In the current study, we used the same KR-TRF1 system to show that endogenous and ectopically expressed MYH, SIRT6, and 9-1-1 are also recruited to oxidatively damaged telomeres, suggesting that BER and DDR are involved in telomere maintenance following oxidative stress.

In this report, we have shown that expressed GFP-tagged MYH and Hus1 form foci in a very similar manner to endogenous proteins, with respect to their colocalization with telomeres. Foci analyses of expressed GFP-tagged proteins or endogenous proteins have some limitations. GFP-tagged proteins are expressed at a higher copy number and need to retain their biological functions. At the same time, detection of endogenous proteins in cells by immunofluorescent staining with their respective antibodies has some drawbacks. First, the specificity of antibodies towards antigens is not always ensured. Second, the staining procedure could indirectly affect consistency of experimental results. Third, antibodies cannot be applied for live cell studies. Thus, the best way to understand the dynamics of proteins in cells is to combine the immunofluorescent staining of endogenous proteins with the direct imaging of tagged proteins in live cells. In our study, we have confirmed foci formation of our studied proteins using antibodies for endogenous proteins (Figures 5E–5G, Supplementary Figures 2E, 2F, and 4) and using GFP-tagged proteins in live cells (Figures 1–4 and 5A–5C). GFP-tagged proteins have been widely used in previous studies to establish the sequential and spatial order of assembly and disassembly of DNA repair proteins [69–71].

Using different knockout cell lines and MYH mutants, we show that MYH recruitment to damaged telomeric chromatin is dependent on Hus1 and SIRT6. The recruitment of Hus1 to damaged telomeres is partially dependent on SIRT6; however, Hus1 recruitment to damaged telomeres is independent of MYH. SIRT6 recruitment to damaged telomeres is independent of both MYH and Hus1. Overall, our results establish that SIRT6 responds very early to damaged telomeres following oxidative stress and suggests that MYH, SIRT6, and 9-1-1 act together to repair oxidative DNA damages within telomeric chromatin. This is supported by our finding that interrupting MYH interactions with its partners by expressing IDC-WT peptide can increase cell’s sensitivity to H_2_O_2_ and elevate cellular 8-oxoG levels after H_2_O_2_ treatment. While expression of IDC-WT peptide impedes MYH interactions with Hus1, SIRT6, and APE1, expression of two mutant IDCs only interrupts certain protein-protein interactions. Expression of IDC-V315A peptide, which interferes with MYH-APE1 interaction, but not MYH-Hus1 and MYH-SIRT6 interactions, affects cell viability but not 8-oxoG levels as compared to cells containing vector alone. This result suggests MYH-APE1 interaction is important in controlling cell viability but not 8-oxoG levels. Expression of IDC-Q324H peptide, which blocks MYH-APE1 and MYH-SIRT6 interactions, but not MYH-Hus1 interaction, exhibits increased cell viability and reduced 8-oxoG levels as compared to expression with IDC-WT peptide. This result suggests that MYH-Hus1 interaction is critical in reducing 8-oxoG levels and also important in controlling cell viability. Because cells expressing IDC-V315A survive better than cells expressing IDC-Q324H, MYH-SIRT6 interaction is important in controlling cell viability. Thus, all three MYH-APE1, MYH-Hus1 and MYH-SIRT6 interactions are important in improving cell viability, but only MYH-Hus1 interaction is critical in reducing 8-oxoG levels when cells are under oxidative stress. Because MYH does not directly remove 8-oxo-G, it is suggested that MYH may convert A/G^o^ to C/G^o^ which is then repaired to C/G by OGG1 or other repair pathways [72]. Therefore, 9-1-1 may coordinate the MYH repair pathway with other repair pathways.

Interestingly, 11-20% of endogenous and over-expressed GFP-tagged MYH and SIRT6 are enriched in telomeres in cells without external acute oxidative stress. However, only 3-7% of GFP-Hus1, endogenous Hus1, and FLAG-Rad9 are colocalized with telomeres in undamaged cells. We suggest that MYH and SIRT6 are responsible for coping with replication stress and repairing endogenous oxidative DNA damage induced by internal sources including inflammation and oxidative phosphorylation at telomeres. At this low level of DNA damage, only a small amount of 9-1-1 is enriched at telomeres indicating DDR is not fully induced. When telomeres are heavily damaged by the KR-TRF1 inducible system, MYH and SIRT6 form foci to repair DNA damage, and 9-1-1 is recruited to activate DDR, which may enhance DNA repair and/or induce cell cycle arrest. In KR-TRF1 induced cells, about 50% of GFP-MYH and 70% of GFP-Hus1 are colocalized with telomeres (Figure 1C and 1I) and about 80% of endogenous mMyh and mHus1 are colocalized with telomeres (Supplementary Figure 4C and 4F). Thus, at least 50% of MYH are colocalized with Hus1 at telomeres. Damages induced by the KR-TRF1 system may produce clustered oxidative damage leading to telomere abbreviation, shortening, and loss. Under this situation, 9-1-1 dependent DDR is induced to arrest the cell cycle or to activate apoptosis [52].

**Figure 7:**
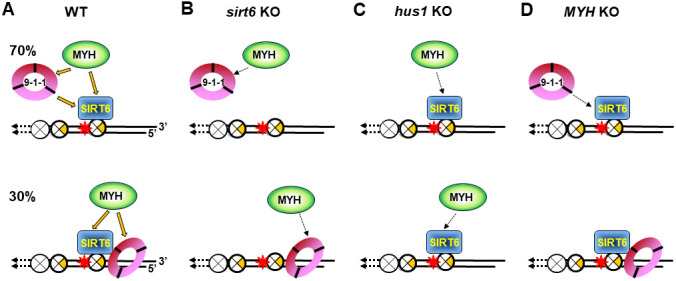
**Models for response of SIRT6, MYH, and Rad9-Rad1-Hus1 to repair DNA damages on damaged telomeres.** SIRT6, MYH, and Rad9-Rad1-Hus1 form a repair complex in a cooperative manner. (**A**) SIRT6 is recruited to damage telomeres at a very early step and then recruits MYH and Hus1 following oxidative stress. However, 30% of Hus1 can also respond to damaged telomeres independently on SIRT6. (**B**) A weaker interaction between MYH and Hus1 in the *sirt6*^-/-^ cells leads to the loss of association of MYH at damaged telomeres. (**C**) A weaker interaction between MYH and SIRT6 in the *hus1*^-/-^ cells leads to the loss of association of MYH at damaged telomeres. (**D**) A weaker interaction between Hus1 and SIRT6 in the *MYH* KO cells reduces the SIRT6-dependent 9-1-1 association but does not affect the SIRT6-independent 9-1-1 association at damaged telomeres.

In the current study, we provide the first evidence that SIRT6 plays an essential role in BER and DDR (Figure 7A). SIRT6 has been shown to participate in BER [38, 41–43]. Our previous results provide a direct functional role of SIRT6 in BER through interaction with MYH, APE1, and the 9-1-1 complex and these interactions are enhanced following oxidative stress [40]. Now, we show that SIRT6 overexpression in oxidatively stressed *sirt6* KO cells can reduce 8-oxoG levels and apoptosis (Supplementary Figure 1B and 1E). It has been reported that SIRT6 responds to DNA double-strand breaks [73] and is one of the enzymes most rapidly recruited at sites of DNA damage [34]. Our data indicate that SIRT6 also acts as an early sensor to recruit MYH and 9-1-1 to the damaged sites. SIRT6 and 9-1-1 can then enhance MYH glycosylase activity [40, 55]. Our results show that the formation of all MYH foci (Figure 3D and 3E) and the majority of Hus1 foci (Figure 4D and 4E) at damaged telomeres is dependent on SIRT6. However, our current data in Figure 3D shows that MYH does not form foci in KR-TRF1 damaged *sirt6*^-/-^ cells, contradicting our previous report that the number of mMyh foci are slightly increased after *sirt6*^-/-^ cells are globally treated with H_2_O_2_ (45% vs. 30% localized on telomeres as compared to untreated *sirt6*^-/-^ cells) [40]. Although the reason is not clear now, the two systems to induce oxidative DNA damage are different. Because <1% of genome is telomeric DNA, localized telomeric DNA damage response may be different from a global ROS-induced response in *sirt6*^-/-^ cells.

Surprisingly, in *sirt6*^-/-^ cells, MYH cannot be recruited to damaged telomeres (Figure 3D and 3E), even though MYH interacts with Hus1, whose foci formation is partially independent of SIRT6 (Figure 4E). Moreover, in *hus1*^-/-^ cells, MYH cannot be recruited to damaged telomeres (Figure 2D and 2E) through interaction with SIRT6 whose foci formation is independent of Hus1 (Figure 4J). These results are probably caused by a weaker interaction between MYH and Hus1 in the *sirt6*^-/-^ cells (Figure 7B) and a weaker interaction between MYH and SIRT6 in the *hus1*^-/-^ cells (Figure 7C). This reasoning is based on our findings that the formation of a physical DNA repair complex consisting of MYH, SIRT6, 9-1-1, and APE1 is cooperative [40, 65]. We have shown that Hus1 enhances the association of APE1 to MYH [65] and that APE1 and Hus1 enhance the association of SIRT6 to MYH [40]. Although Hus1 and SIRT6 bind to the IDC of MYH, there is no competition between SIRT6 and Hus1 binding to MYH. Instead, we show that Hus1 enhances the association of SIRT6 to MYH [40]. Thus, when one partner is absent, the DNA damage response of other factors is affected.

The molecular mechanism(s) responsible for how 9-1-1 enhances and coordinates BER remains elusive. It has been suggested that 9-1-1 is loaded onto RPA-coated single-stranded DNA to activate DDR [19, 74]. Here, we show that about 70% of Hus1 foci formation at damaged telomeres are dependent on SIRT6 (Figure 4E). We propose that 30% of SIRT6-independent Hus1 foci can form at 5’recessed telomeric DNA ends because 9-1-1 is preferentially loaded onto DNA with 5’ recessed ends [75–78]. This may explain why about 40% of Hus1 and Rad9 foci expanded unidirectionally at KR-TRF1 sites (Figure 1M and 1N). However, the significance of Hus1 and Rad9 foci expanding from the site of KR-TRF1 requires further examination. Our data indicates that 9-1-1 is required for MYH recruitment to damaged telomeric chromatin, but Hus1 foci formation at damaged telomeric chromatin is independent of MYH. These results appear to be contradictory to our previously published results that deletion of SpMyh1 reduces, but does not completely prevent SpHus1 association to *S. pombe* telomeres by chromatin immunoprecipitation (ChIP) assay [79]. The difference between these two studies is that the current study uses a fluorescence method to detect quantitative colocalization of protein foci at telomeres and the other uses ChIP assay to measure the qualitative folds of protein enrichment at telomeres. As such, the amount of Hus1 recruited to telomeres may be reduced while the percentage of Hus1 foci may remain the same. As discussed above, because Hus1 forms a complex with MYH and SIRT6 [40], we suggest that Hus1 association at damaged telomeres may be lessened by weaker interactions between SIRT6 and 9-1-1 in *MYH* KO cells (Figure 7D).

The ordered assembly of MYH, SIRT6, and 9-1-1 at oxidatively damaged telomeres may ensure that DNA repair, chromatin remodeling, and DDR are coordinated. Interestingly, we observed that the catalytic activities of SIRT6 are not important for SIRT6 response but are essential for MYH recruitment to damaged telomeres. Because SIRT6^H133Y^ mutant has been shown to lack both NAD^+^-dependent protein deacetylation and ADP-ribosylation [38, 41] and also has very weak defatty-acylase activity [64], it remains to be examined which SIRT6 activity plays this essential role. Our previous data suggest that SIRT6 in complex with MYH, APE1, and 9-1-1 at sites of DNA damage may undergo auto mono-ADP-ribosylation, leading to altered chromatin structure and optimal DNA repair [40]. Also, SIRT6 mono-ADP ribosylates PARP1 and KDM2A lysine demethylase at DNA damage sites to promote repair [41, 80]. These findings suggest that mono-ADP-ribosylase activity of SIRT6 may be important for recruiting BER factors. On the other hand, the deacetylase activity of SIRT6 may enhance MYH repair through nucleosome remodeling. It is interesting to note that SIRT6 recruits SNF2H to DNA break sites [34] and to KR-TRF1 damaged telomeres [39], causing locally decondensed chromatin. However, the SNF2H recruitment by SIRT6 is independent of SIRT6 catalytic activities [39]. Further studies are needed to assess the mechanisms underlying SIRT6 recruitment and SIRT6’s roles in BER following DNA damage.

## MATERIALS AND METHODS

### Cell culture

HEK-293T cells [from American Type Culture Collection (ATCC)] were maintained in DMEM (Thermo Fisher Scientific) supplemented with 4.5 mg/ml D-glucose, 2 mM L-glutamine, 1 mM sodium pyruvate, and 10% fetal bovine serum. *Sirt6*^+/+^ (wild-type, WT) and *sirt6*^-/-^ (KO) mouse embryonic fibroblast (MEF) cells (obtained from Dr. Raul Mostoslavsky at Harvard Medical School) were maintained in DMEM supplemented with 15% fetal bovine serum and 1% Penicillin-Streptomycin. CT2 (*hus1^+^*^/+^) and CT7 (*hus1*^−/−^*p21*^−/−^) MEF cells (obtained from Dr. Robert Weiss at Cornell University) were maintained in media as described for *Sirt6*^+/+^ and *sirt6*^-/-^ MEF cells.

The *hMYH* gene in HEK-293T cells was knocked out by CRISPR-Cas9 method [62]. gRNA (MUTYH CRISPR Guide RNA 2 or crRNA 2) with sequence ACTGTGATCAACTACTATAC (located in Exon 5 of *hMYH* gene) in PX459 plasmid [81] was purchased from GenScript. MYH crRNA 2 was transfected into HEK-293T cells followed by 1.25 μg/ml puromycin selection and single colonies were screened, expanded, and confirmed by DNA sequencing and Western Blot analysis.

### Plasmids

Plasmids pEGFP-*hMYH* [40], pEGFP-*hSIRT6* [40], and pEGFP-*hSIRT6* encoding catalytically inactive SIRT6^H133Y^ mutant [39] have been described. pCMV KR-TRF1, pCMV DsRed-TRF1, and pCMV RFP-TRF1 plasmids have been described [52]. pEGFP-*hMYH^V315A^* and pEGFP-*hMYH^Q324H^* were generated by site-directed mutagenesis with primers listed in Supplementary Table 1. *hHus1* were subcloned by PCR amplification using template pET21a-*hHus1* [55] and primers listed in Supplementary Table 1. The PCR product of *hHus1* was digested with BamHI and SalI and ligated into the BamHI-SalI digested pEGFP-C1 vector (Clonetech Laboratories). FLAG-hRad9 [60] was obtained from Dr. Mihoko Kai at Johns Hopkins University. GFP-tagged IDC peptides (residues 295-350) with wild-type, V315A, or Q324H sequences were cloned using plasmids containing the full length wild-type and mutant *hMY*H genes [40] as templates, utilizing the primers listed in the table, into pEGFP-N1 (Clonetech Laboratories). A nuclear localization signal sequence was added at the C-terminus of the IDC peptide.

### Transfections

For confocal microscopy studies, plasmids were transfected with Lipofectamine2000 (Thermo Fisher Scientific) using a standard protocol. For cell viability and 8-oxoG assays, plasmids were transfected into mammalian cells using X’tremeGENE HP transfection reagents (Millipore-Sigma, 6366244001). Transiently transfected cells were used for all analyses.

### Confocal microscopy and image quantification

Twenty four or 36 hours after transfection with plasmids, cells were exposed to 15-W SYLVANIA cool white fluorescent bulbs for 30 minutes in a stage UVP (Uvland) to induce DNA damage as described [52, 82]. Cells were cultured for various times after white light exposure. All cells were then fixed with 4% (vol/vol) formaldehyde for 15 min at room temperature (RT) and subsequently washed 3 times with PBS. The images were acquired using the Olympus FV1000 confocal microscopy system (Cat. F10PRDMYR-1, Olympus) and FV1000 software. For calculation of the percentage of GFP-fusion proteins colocalized with KR-TRF1, 20 cells were counted manually. To avoid the interference of background signal, foci with fold increase of mean intensity of the protein foci at telomere/non-telomere background larger than 1.3 are defined as positive foci. Three independent experiments were performed, and representative data are shown. Fluoview Soft (Olympus) was used for data analysis.

### Immunofluorescence staining

After cells were transfected with plasmids and exposed to light and recovered as described above, they were fixed with 3.7% (v/v) formaldehyde for 15 min at RT, followed by three washes with PBS. Cell were then permeabilized with PBS containing 0.2% Triton X-100 for 5 min at RT and washed with PBS twice. After being blocked in PBS containing 15% fetal bovine serum for 15 min at 37°C, the cells were reacted with polyclonal antibodies against an hMYH peptide (α344) [83] or hHus1 monoclonal antibody (Novus, NBP1-89445) overnight at 4°C. Next, the cells were washed three times for 15 min each in PBS and incubated with Alexa Fluor® 594 goat anti-rabbit secondary antibodies (Invitrogen) diluted in DMEM + Azide for 30 min at RT. Cell samples were then mounted in drops of PermaFluor (Immunon). Image quantification was analyzed as described in section 2.4.

To analyze endogenous mMyh foci in *sirt6* KO MEF cells expressing GFP-SIRT6 and Myc-tagged KR-TRF1, immunofluorescence staining procedures were modified as follows. The *sirt6* KO MEF cells were transfected with pEGFP-C1 vector (Clonetech Laboratories) (or pEGFP-C1-SIRT6) and pCMV KR-TRF1. After fixing with formaldehyde, cells were incubated with 2.5M HCl for 1 min to strip the green and red fluorescence. Cells were then permeabilized with PBS containing 0.2% Triton X-100 for 10 min followed by incubating with 5% bovine serum albumin for one hour. Next, the cells were reacted with primary antibodies of Myc (mouse monoclonal, ab32, Abcam), MYH (Rabbit polyclonal, α344), GFP (chicken polyclonal, ab13970, Abcam) overnight. After being washed three times for 5 min each with PBS, the cells were incubated with secondary antibodies (goat anti-mouse, Fluor®594, ab150116; goat anti-rabbit, Fluor®488, ab150077; goat anti-chicken Fluor®405, ab175674) for one hour and then followed by three washes times for 5 min each with PBS.

### Cell viability analysis

Cell viability was measured using the neutral red uptake assay as described [72]. Cells were seeded in 12-well flat bottom tissue culture plates. One day post-seeding, the cells were treated with 700 mM H_2_O_2_ for 1 h. After recovery in fresh medium for two days, the plates were incubated for 2 h in regular medium containing 40 μg/ml of neutral red (3-amino-7-dimethylamino-2-methyl-phenazine hydrochloride, Sigma). The cells were then washed with PBS twice; the dye was extracted from each well with acidified ethanol solution. Plates were incubated at room temperature with gentle shaking for 15 minutes and the absorbance at 540 nm was read in a Multiskan Spectrum microplate spectrometer (Thermo Scientific).

### Quantification of 8-oxo-G

Cells were seeded on a 2-chamber culture slide, treated with 700 mM H_2_O_2_ for 1 h and recovered for 1 h in fresh media. Cells were fixed in 1:1 methanol:acetone for 20 min at −20°C followed by 0.05 N HCl treatment for 5 min. RNA was removed by 100 μg/ml RNase A in 150 mM NaCl and 15 mM sodium citrate for 1 h at 37°C and DNA was denatured in situ with 0.15 N NaOH in 70% ethanol for 4 min. Cells were then treated with 5 μg/ml proteinase K in 20 mM Tris–HCl (pH 7.5) and 1 mM EDTA for 10 min at 37°C and blocked with 5% normal goat serum in PBS at room temperature for 1 h. Cells were then incubated with anti-8-oxo-dG antibody (Trevigen, 4354-MC-050) in PBS containing 1% BSA at 4°C overnight. Next, the cells were washed three times for 5 min each in PBS and incubated with Alexa Fluro 592 donkey anti-mouse antibodies (Molecular Probes) at a 1:250 dilution in PBS containing 1% BSA for 1 h at room temperature at dark. The slides were then washed three times in PBS with 0.05% Tween-20. Nuclear DNA was counterstained with 5 μg/ml DAPI. Slides were mounted with cover slip using mounting medium (Leica micromount) and images were captured with DMi8b fluorescent microscope (Leica).

## Supplementary Material

Supplementary Methods

Supplementary Figures

Supplementary Table 1
