# Supplementary material for: An ordered assembly of MYH glycosylase, SIRT6 protein deacetylase, and Rad9-Rad1-Hus1 checkpoint clamp at oxidatively damaged telomeres
Source: Aging (Albany NY). 2020 Sep 29;12(18):17761–85. doi: 10.18632/aging.103934 (PMC7585086; doi:10.18632/aging.103934)
Supplement: Supplementary Methods [file aging-12-103934-s001..pdf]

SUPPLEMENTARY METHODS

Western blotting

To quantitate the expression levels of GFP-tagged proteins, cell extracts of wild-type, knockout, and wild-type transfected with GFP-plasmids were prepared from cells grown to late log phase and compared with western blotting. Cells from one 10 cm dish (~1 × 10^7cells) were lysed in 0.3–0.5 ml of RIPA buffer (50 mMTris–HCl, pH 7.4, 150 mM NaCl, 1% NP40, 1 mM EDTA, 0.1% TritonX-100, 1 mM phenylmethylsulfonyl fluoride, 1 mM NaF, and 1 mM Na_3VO_4) followed by incubation at 4°C for 30 min followed by centrifugation at 14,000 rpm for 10 min. The supernatant was aliquoted and stored at −80°C. The protein concentration was determined by Bio-Rad protein assay (Bio-Rad). Proteins were fractionated by SDS-polyacrylamide gels and transferred to a nitrocellulose membrane. The membranes were allowed to react with antibodies against an hMYH peptide (α344) [1], hHus1 (sc-8323, Santa Cruz Biotechnology), hSIRT6 (Sigma), or actin (Sigma). Western blotting was detected by the Enhanced Chemiluminescence (ECL) analysis system (GE Health) according to the manufacturer's protocol. Signals were detected by GE-Amersham Imager 680 RGB.

Colony formation (clonogenic survival) analysis

Clonogenic survival was measured as described [2]. Cells (about 1,000 cells) were seeded in 6-well culture plates. One day post-seeding, the cells were transfected with plasmid and then treated with hydroxyurea for 2 days or left untreated. Cells were washed with PBS twice and recovered in fresh media. After 7 days, cells were stained with 0.5% crystal violet in 20% methanol. After 30 min, plates were washed with water to remove the background color and allowed to air dry. Images were then scanned and analyzed.

Apoptosis TUNEL assay

The apoptotic cells were detected by terminal deoxynucleotidyl transferase-mediated dUTP nick-end labeling (TUNEL) assay in accordance with the manufacturer's protocol (Promega) [2]. Briefly, cells were seeded on a 2-chamber culture slide (Quality Biological, 229162) and treated with H_2O_2 in serum-free media for 1 hour and recovered for 2 h in fresh media. Cells were fixed in 4% paraformaldehyde in PBS for 15 minutes on ice and then treated with 20 µg/ml proteinase K in 20 mM Tris-HCl (pH 8.0) and 50 mM EDTA for 5 minutes at room temperature. Slides were then immersed in Coplin jars containing PBS for five minutes at room temperature. Cells were then washed with 100 µl of wash buffer (Abcam, ab66110) for 5 minutes. Next, cells were covered in DNA Labeling solution (Abcam, ab66110) and incubated at 37°C in the dark for 1 hour. Slides were then washed 2 times for 5 minutes in PBS. Chambers were then incubated with 100 µl each of Antibody Solution (Abcam, ab66110) for 30 minutes at room temperature in the dark. Slides were then washed once in ddH_2O for 5 minutes. Nuclear DNA was then counterstained with 5 µg/ml DAPI. Slides were washed 2 times in ddH_2O. Slides were mounted with cover slips using mounting medium (Leica micromount) and images were captured with DMi8b fluorescent microscope (Leica).

SUPPLEMENTARY REFERENCES

1. Parker A, Gu Y, Lu AL. Purification and characterization of a mammalian homolog of Escherichia coli MutY mismatch repair protein from calf liver mitochondria. Nucleic Acids Res. 2000; 28:3206–15. https://doi.org/10.1093/nar/28.17.3206 PMID:10954587
2. Hwang BJ, Shi G, Lu AL. Mammalian MutY homolog (MYH or MUTYH) protects cells from oxidative DNA damage. DNA Repair (Amst). 2014; 13:10–21. https://doi.org/10.1016/j.dnarep.2013.10.011 PMID:24315136
