# Supplementary material for: An ordered assembly of MYH glycosylase, SIRT6 protein deacetylase, and Rad9-Rad1-Hus1 checkpoint clamp at oxidatively damaged telomeres
Source: Aging (Albany NY). 2020 Sep 29;12(18):17761–85. doi: 10.18632/aging.103934 (PMC7585086; doi:10.18632/aging.103934)
Supplement: Supplementary Figures [file aging-12-103934-s003..pdf]

SUPPLEMENTARY FIGURES

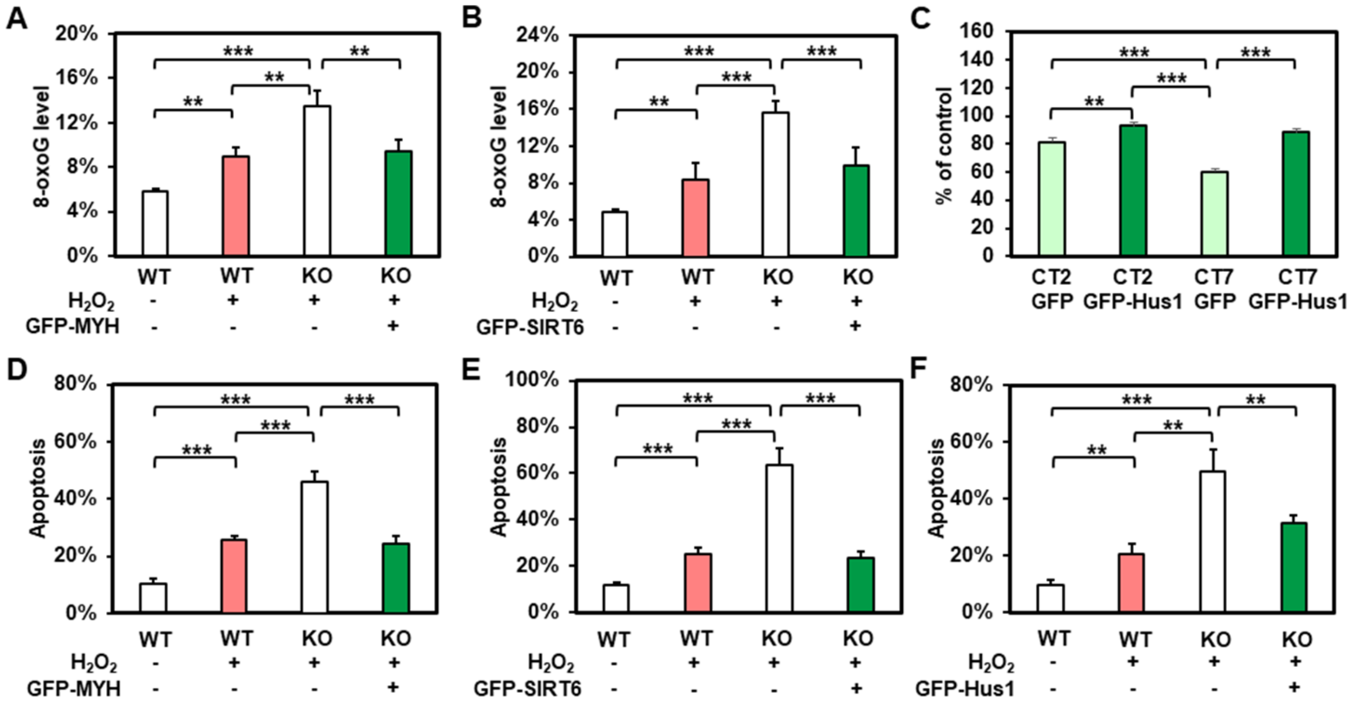

Supplementary Figure 1. Functional assays of GFP-proteins expressed in knockout cells. (**A**) 8-oxoG levels of HEK-293T wild-type (WT) and HEK-293T *MYH* KO cells transfected with GFP-hMYH plasmid with or without H_2O_2 treatment. (**B**) 8-oxoG levels of WT-MEF and *Sirt6* KO cells transfected with GFP-hSIRT6 plasmid with or without H_2O_2 treatment. (**C**) Sensitivity to 1 mM of hydroxyurea of Hus1 WT (CT2) and *hus1*$^{-/-}$ (CT7) cells expressing GFP vector or GFP-hHus1. (**D**) Percentages of apoptosis of HEK-293T WT and *MYH* KO cells transfected with GFP or GFP-hMYH plasmid with or without H_2O_2 treatment. (**E**) Percentages of apoptosis of WT-MEF and *Sirt6* KO cells transfected with GFP or GFP-hSIRT6 plasmid with or without H_2O_2 treatment. (**F**) Percentages of apoptosis of WT-MEF and *Hus1* KO cells transfected with GFP or GFP-hHus1 plasmid with or without H_2O_2 treatment. Error bars indicate SD; n ≥ 3. The *P*-value is calculated by Student's t-test using Stat Plus software; *, **, and *** represent *P <0.1, P <0.05, and P* < 0.01, respectively.

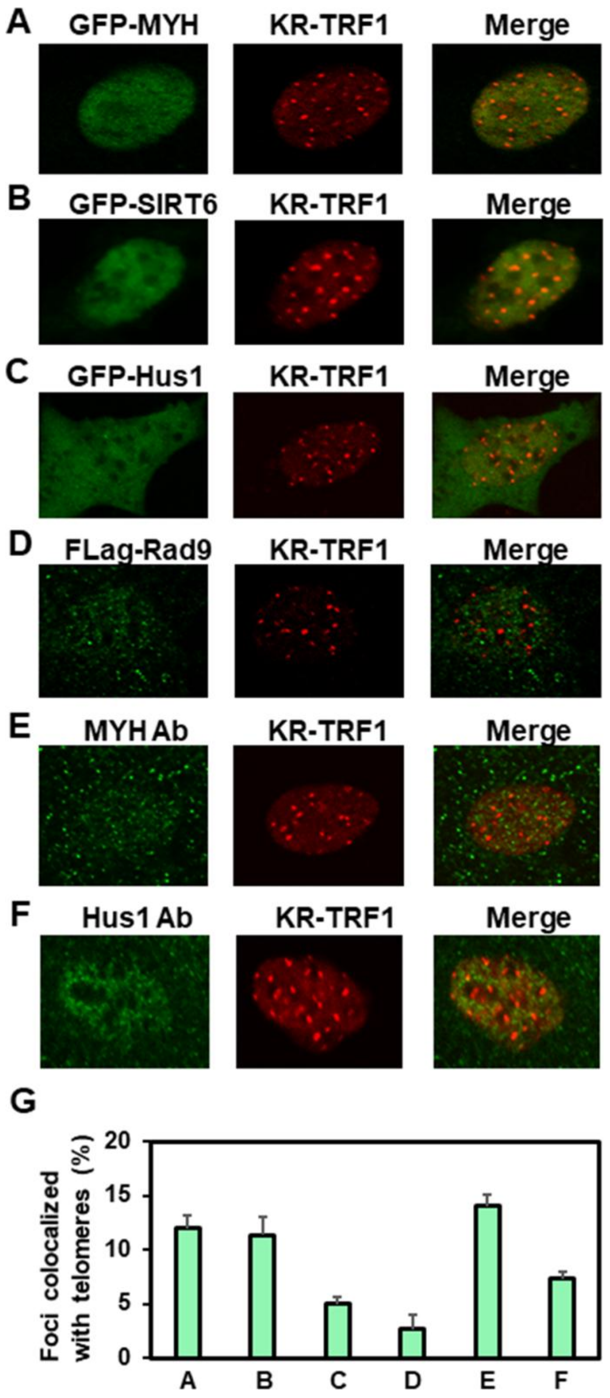

Supplementary Figure 2. Distribution of repair proteins in cells containing un-induced KR-TRF1. The experiments and analyses are similar to those as described in Figures 1 and 2 except KR was not induced. (**G**) Quantitative analyses of granules colocalized to telomeres. Twenty cells in each un-induced KR-TRF1 group (**A**–**F**) were analyzed. Error bars indicate SD.

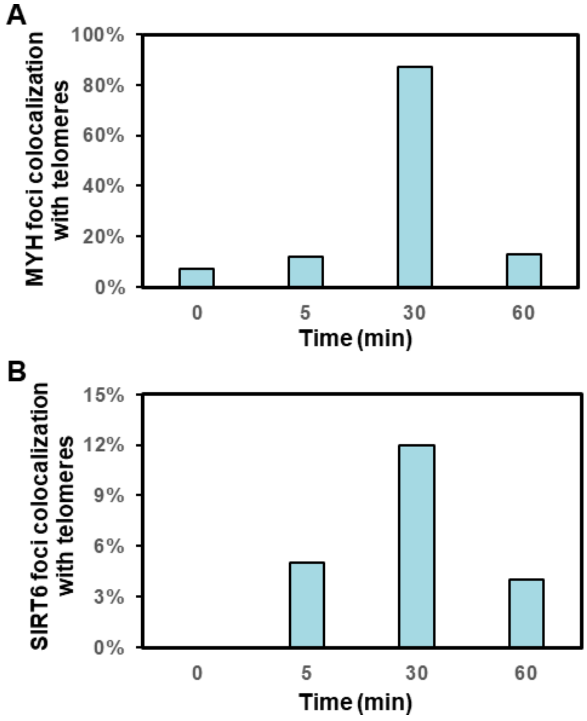

Supplementary Figure 3. Kinetics of GFP-tagged MYH (**A**) and SIRT6 (**B**) response to DNA damage at telomeres. After transfection with plasmids, cells were exposed to white fluorescent for 30 minutes to induce DNA damage. Cells were cultured for various times (as indicated) after white light exposure. Confocal microscopy and image quantification are described as MATERIALS AND METHODS.

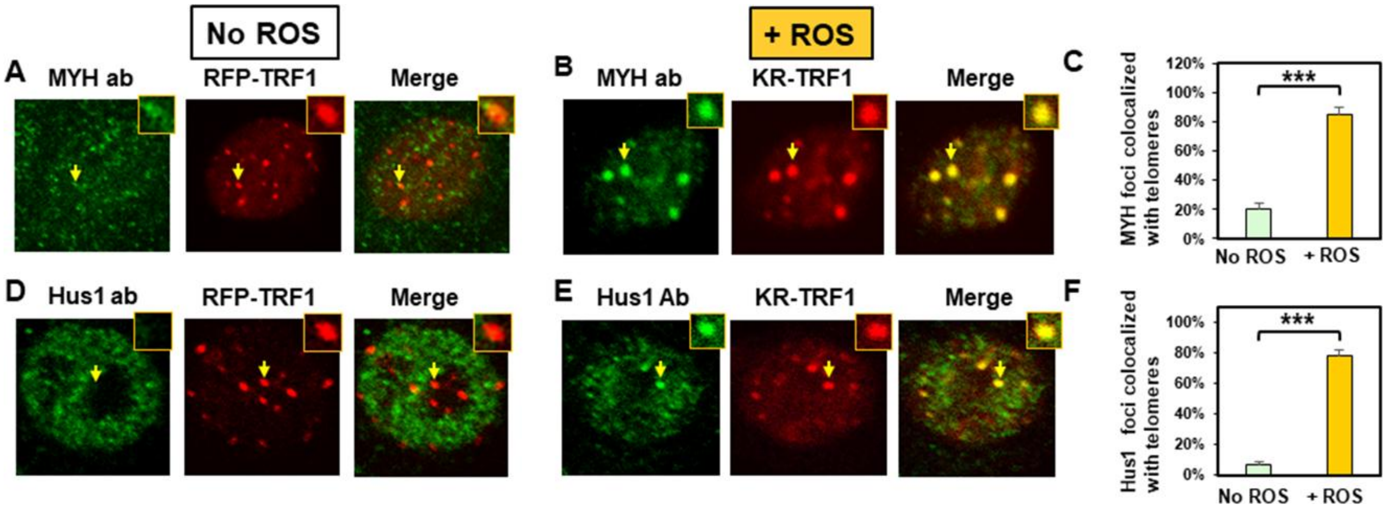

Supplementary Figure 4. Endogenous MYH and Hus1 are recruited to oxidatively damaged telomeric sites in mouse embryonic fibroblast (MEF) cells. MYH and Hus1 were detected by immunofluorescence staining. (**A** and **D**), Distribution of hMYH and hHus1, respectively, in undamaged MEF cells containing RFP-TRF1. (**B** and **E**), Distribution of hMYH and hHus1 foci, respectively, at KR-TRF1 damaged telomeric sites after light activation. Images were captured 30 min after light activation with an Olympus FV1000 confocal microscopy system. (**C** and **F**), Quantitative analyses of 20 cells in each undamaged and KR-induced damaged group.

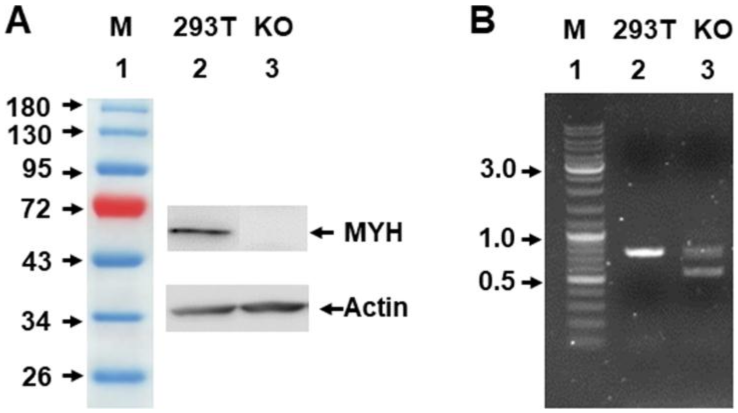

Supplementary Figure 5. Analysis of 293T MYH KO clones. (**A**) Western blotting of extracts from parental HEK-293T (lane 2) and MYH knockout (KO) HEK-293T (clone 7a) (lane 3) cells with hMYH and β-actin antibodies. Lane 1, protein molecular markers with marked molecular weight in KDa. (**B**) Agarose gel analysis of PCR products with primer Chang594 and Chang 638 from parental HEK-293T (lane 2) and MYH knockout (KO) HEK-293T (clone 7a) (lane 3) cells. Lane 1, DNA size markers in Kb. The upper and lower bands from KO cells were subject for DNA sequencing with primers Chang639 and Chang640 that indicates that the upper band contains an adenine insertion at 45333112 and the lower band contains a 229-bp deletion at 45333114-45333341 as indicated in genomic reference # NC_000001.11.
