# Supplementary material for: An ordered assembly of MYH glycosylase, SIRT6 protein deacetylase, and Rad9-Rad1-Hus1 checkpoint clamp at oxidatively damaged telomeres
Source: Aging (Albany NY). 2020 Sep 29;12(18):17761–85. doi: 10.18632/aging.103934 (PMC7585086; doi:10.18632/aging.103934)
Supplement: Supplementary Table 1 [file aging-12-103934-s002..pdf]

SUPPLEMENTARY TABLE

Supplementary Table 1. Primers used for mutagenesis, subcloning, PCR analyses, and DNA sequencing.

Name	Sequence
hMYH ^{V315A} Sense	AGCACACTCCTCCGCGTCAGGACTGCC
hMYH ^{V315A} Anti-sense	GGCAGTCCTGACGCGGAGGAGTGTGCT
hMYH ^{Q324H} Sense	GTGCTCCCAACACTGGACATTGCCACCTGT
hMYH ^{Q324H} Anti-sense	ACAGGTGGCAATGTCCAGTGTTGGGAGCAC
pEGFP-C1 hHus1 Forward	TCT GCA GTC GAC ATG AAG TTT CGG GCC AAG
pEGFP-C1 hHus1 Reverse	CC GGT GGA TCC CTA GGA CAG CGC AGG GAT GAA
Chang 594	AGC CGG AAG AGG TGG TAT TG
Chang 638	CCG GCC ACG AGA ATA GTA GC
Chang 639	TTGCATTGACAGGCAGAAGA
Chang 640	TCACCCGTCAGTCCCTCTAT
IDC-F	AGATCTCGAGATGCGCCAGAGAGTGGAGCAGGAACAG
IDC-R	CTGCAGAATTCGATCTAGCTTAACTCTCTTAGCAGCTGGGGGGGGCTTGCG GCTGGCCT
